# Characterization of MOSFET dosimeters for low‐dose measurements in maxillofacial anthropomorphic phantoms

**DOI:** 10.1120/jacmp.v16i4.5433

**Published:** 2015-07-08

**Authors:** Juha H. Koivisto, Jan E. Wolff, Timo Kiljunen, Dirk Schulze, Mika Kortesniemi

**Affiliations:** ^1^ Department of Physics University of Helsinki Helsinki Finland; ^2^ Department of Oral and Maxillofacial Surgery/Pathology VU University Medical Center Amsterdam The Netherlands; ^3^ International Docrates Cancer Center Helsinki Finland; ^4^ Dental Diagnostic Center Freiburg Germany; ^5^ HUS Helsinki Medical Imaging Center University of Helsinki Helsinki Finland

**Keywords:** MOSFET dosimeter, X‐ray, radiation exposure

## Abstract

The aims of this study were to characterize reinforced metal‐oxide‐semiconductor field‐effect transistor (MOSFET) dosimeters to assess the measurement uncertainty, single exposure low‐dose limit with acceptable accuracy, and the number of exposures required to attain the corresponding limit of the thermoluminescent dosimeters (TLD). The second aim was to characterize MOSFET dosimeter sensitivities for two dental photon energy ranges, dose dependency, dose rate dependency, and accumulated dose dependency. A further aim was to compare the performance of MOSFETs with those of TLDs in an anthropomorphic phantom head using a dentomaxillofacial CBCT device. The uncertainty was assessed by exposing 20 MOSFETs and a Barracuda MPD reference dosimeter. The MOSFET dosimeter sensitivities were evaluated for two photon energy ranges (50–90 kVp) using a constant dose and polymethylmethacrylate backscatter material. MOSFET and TLD comparative point‐dose measurements were performed on an anthropomorphic phantom that was exposed with a clinical CBCT protocol. The MOSFET single exposure low dose limit (25% uncertainty, k=2) was 1.69 mGy. An averaging of eight MOSFET exposures was required to attain the corresponding TLD (0.3 mGy) low‐dose limit. The sensitivity was 3.09±0.13 mV/mGy independently of the photon energy used. The MOSFET dosimeters did not present dose or dose rate sensitivity but, however, presented a 1% decrease of sensitivity per 1000 mV for accumulated threshold voltages between 8300 mV and 17500 mV. The point doses in an anthropomorphic phantom ranged for MOSFETs between 0.24 mGy and 2.29 mGy and for TLDs between 0.25 and 2.09 mGy, respectively. The mean difference was −8%. The MOSFET dosimeters presented statistically insignificant energy dependency. By averaging multiple exposures, the MOSFET dosimeters can achieve a TLD‐comparable low‐dose limit and constitute a feasible method for diagnostic dosimetry using anthropomorphic phantoms. However, for single *in vivo* measurements (<1.7 mGy) the sensitivity is too low.

PACS number: 87.50.wj

## I. INTRODUCTION

Monitoring of the radiation exposure induced by dental examinations has become more important due to the rapidly increasing number of X‐ray devices in use. Cone‐beam computed tomography (CBCT) devices are becoming more commonly used and cause substantially higher patient doses^(1)^ when compared with conventional dental radiography devices.

To date, most studies concerning effective dose assessment have used thermoluminescent dosimeters (TLD) in combination with anthropomorphic phantoms.^2^, ^3^, ^4^, ^5^, ^6^, ^7^ The low‐dose limit of TLDs when using single exposure (0.3 mGy)^8^, ^9^ has been considered adequate in most applications and has, therefore, been used as the reference low‐dose limit in MOSFET uncertainty assessment in this study. The main drawback with using TLDs is that they need to be replaced and read after every exposure. This can become tedious and time‐consuming when a series of different measurements are performed.

One possible alternative to TLDs is Metal Oxide Semiconductor Field Effect Transistor (MOSFET) dosimeters that can be used for near real‐time, point‐dose measurements in anthropomorphic phantoms. Until recently, MOSFETs were mainly used in radiotherapy,^10^, ^11^, ^12^, ^13^ but are now being more commonly used in different fields of diagnostic radiology.^14^, ^15^, ^16^, ^17^, ^18^ A drawback with MOSFET dosimeters is that they have limited sensitivity when compared with TLD dosimeters.

To date, most studies focusing on MOSFET characterization of diagnostic radiology use TLDs.^9^, ^13^, ^19^ MOSFET dosimeters should ideally be characterized similarly to TLDs, and calibrated at the radiation qualities close to those for which measurements are needed.^(20)^ A more recent study by Manninen et al.^(21)^ evaluated MOSFET dose linearity, repeatability and sensitivity using tube voltages between 40–125 kVp. They used radiophotoluminescence dosimeters (RPLD) instead of TLDs to characterize MOSFET (TN‐1002RD) dosimeters that have different attenuation properties than the reinforced dosimeter type (TN‐1002RD‐H) used in this study. In their study, Manninen et al. focused on characterizing MOSFETs, using two different conventional radiographic devices (Philips Optimus 50, Siemens Axion Aristos FX) and with different aluminum filters.

Low‐dose inaccuracies can have a negative effect on the outcome of the measurements when using MOSFETs for diagnostic purposes. The main cause for the low‐dose inaccuracies is type A (statistical) uncertainty that can be improved by increasing the number of exposures and subsequently averaging the results.^(22)^ However, multiple exposures can only be performed on anthropomorphic phantoms and not as *in vivo* measurements on patients.

This study has three main aims. The first aim was to characterize the MOSFET dosimeter uncertainty and to calculate the single exposure low dose limit and to subsequently determine the number of exposures required to attain (25% uncertainty, k=2) the TLD comparable (0.3 mGy) low‐dose limit. A second aim was to characterize MOSFET dosimeter sensitivities for two energy ranges using a dental and maxillofacial CBCT device. The mean photon energies were attained using two different filter combinations. Furthermore, the MOSFET dosimeters were characterized for dose dependency, dose rate dependency, and accumulated dose dependency. The final aim was to compare the point‐dose measurement performance of MOSFETs with those of TLDs in an anthropomorphic phantom using dental and maxillofacial CBCT imaging.

## II. MATERIALS AND METHODS

### A. Scanner

All measurements were performed using a ProMax 3D MID dental cone‐beam computed tomography (CBCT) device (Planmeca, Helsinki, Finland). The radiation source contained a Toshiba D‐054 X‐ray tube (Toshiba Electron Tubes & Devices Co., Ltd. Otawara‐shi, Japan) with tungsten (W) target, 5° anode angle, 0.5 mm Al‐equivalent inherent filtration, and a 0.5 mm focal spot size. The tube potentials ranged from 50 kVp to 90 kVp, with 5 kV increments, to encompass the typical energy ranges used in dental diagnostic procedures. MOSFET sensitivities were assessed using two different filter combinations (2.5 mm Al, and 2.5 mm Al+0.5 mm Cu) to reproduce two different commonly used dental energy spectra. The X‐ray spectra, expressed in terms of the half value layer (HVL) and the average photon energy (Table 1) were calculated using a computer program^(23)^ that was based on the semi‐empirical spectrum model described by Birch and Marshall.^(24)^

**Table 1:** The calculated mean photon energies and corresponding Cu HVLs, using 2.5 mm Al filter and 2.5 mm AL+0.5mm Cu filter combinations

*Tube Voltage kV*	*Al (2.5 mm)*		Al (2.5 mm)+Cu (0.5 mm)	
	*Mean E keV*	*HVL Cu (mm)*	*Mean E keV*	*HVL Cu (mm)*
50	34.2	0.072	41.2	0.161
55	36.4	0.081	44.2	0.192
60	38.6	0.093	46.9	0.224
65	40.7	0.104	49.6	0.258
70	42.8	0.115	52.2	0.294
75	45.0	0.129	54.7	0.333
80	47.1	0.144	57.1	0.373
85	49.0	0.159	59.2	0.411
90	50.8	0.176	61.1	0.448

### B. Dosimeters

#### B.1 MOSFET reader system

The TN‐RD‐70‐W20 MOSFET device comprises a TN‐RD‐38 wireless Bluetooth transceiver, four TN‐RD‐16 reader modules, twenty high‐sensitivity TN‐1002RD‐H dosimeters, and TN‐RD‐75M software (Best Medical, Ottawa, ON, Canada). In this study, TN‐RD‐16 reader modules that can be independently set to control five dosimeters were operated using the high bias voltage, 13.6 V, to obtain the best possible accuracy. A TN‐RD‐38 Bluetooth transceiver was used for data communication between the TN‐RD‐16 reader modules and a PC.

#### B.2 MOSFET dosimeters

**Figure 1:**
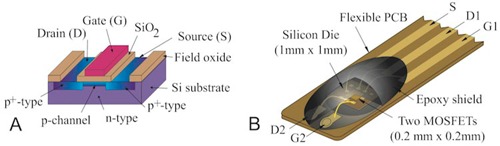
p‐channel MOSFET dosimeter (a) structure and TN‐1002RD‐H dosimeter (b).

MOSFET dosimeter (TN‐1002RD‐H) comprises an n‐type semiconductor substrate that is isolated from the metal gate (G) by a very thin silicon oxide (SiO_2_) layer (Fig. 1(a)). Ionizing radiation causes the generation of electron hole pairs in the ${\text{SiO}}_{2}$ layer. Some of the holes near the Si‐SiO_2_ interface are trapped causing a stable negative shift (${\text{DV}}_{\text{TH}}$) for a predetermined drain (D) – source (S) current ($I_{\text{DS}}$) that is proportional to the radiation dose. The MOSFET dosimeter operating principles have been reported by several investigators.^8^, ^25^, ^26^ The TN‐1002RD‐H dosimeter used in this study comprised two MOSFETs with an active area 0.04 mm^3^ that were fabricated on a silicon rectangle (a die) mounted on a flexible polyamide (PCB) cable and encapsulated with black 1.02 mm thick^(21)^ epoxy resin (Fig. 1(b)).

#### B.3 TLD dosimeters

Forty TLD thermoluminescent dosimeters (TLD 100; Thermo Fisher Scientific, Waltham, MA) were used to compare their performance with MOSFETs in an anthropomorphic RANDO phantom (The Phantom Laboratory, Salem, NY). The reset and annealing procedures of the TLDs were made in a microprocessor‐controlled TLD oven (PTW, Freiburg, Germany). The TLD read‐out sequence was performed in a Fimel LTMWin (Fimel, Fontenay‐aux‐Roses, France) device. TLD calibration and read‐out procedures were performed according to a method previously described by Rottke et al.,^(7)^ with traceability to secondary standard dosimetry laboratory (SSDL, Freiburg, Germany).

#### B.4 Reference dosimeters

A Barracuda multipurpose detector (RTI Electronics AB, Mölndal, Sweden) with a measurable dose range between 0.1μGy−1000 Gy and less than ±1% energy sensitivity variation between 50–90 kVp was used as a reference dosimeter. Reference readings of all energy sensitivity measurements were attained using a RADCAL 1015 dosimeter and a RADCAL 10X5‐6 ionization (Radcal Corporation, Monrovia, CA) chamber with less than ±1% energy sensitivity variation in the 30–70 keV range used in this study. Prior to the measurements, both the Barracuda MPD and the RADCAL 1015 dosimeters were calibrated at the secondary standard dosimetry laboratory (SSDL) of the Finnish Radiation and Nuclear Safety Authority (STUK) that is traceable to primary standard dosimetry laboratory (PSDL).

### C. Phantom

**Figure 2:**
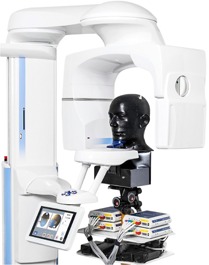
The MOSFET measurement system with four TN‐RD‐16 reader modules, RANDO phantom, and ProMax 3D MID CBCT device.

In the second part of the study, an anthropomorphic RANDO RAN102 male head phantom (Fig. 2) (Radiation Analogue Dosimetry System; The Phantom Laboratory) was used for the comparative dose measurements between TLD and MOSFET dosimeters. The phantom represents an adult male that comprises a human skull embedded in a soft tissue‐equivalent synthetic material to match the attenuation and scattering properties of the bone, soft tissues, and airways of the human head. The phantom was transected into ten 2.5 cm thick layers each comprising a 1.5 cm×1.5 cm grid of φ0.5 cm holes filled with removable, soft tissue‐equivalent plugs for dosimeter placement.

### D. Measurements

#### D.1 MOSFET dosimeter uncertainty

In this study, the MOSFET dosimeter uncertainty was assessed according to the UK National Dose protocol,^(27)^ where the maximum patient dose uncertainty has been proposed to be 25% at 95% confidence level. In order to evaluate the uncertainty, all MOSFET and Barracuda dosimeters were simultaneously exposed to 80 kVp (47.1 keV, HVL 7.71 mm Al, SID 600 mm) using a Promax 3D CBCT device (Planmeca Oy, Helsinki, Finland) with a 2.5 mm Al filter. The X‐ray source was operated without gantry rotation, using seven mAs values (10, 20, 40, 80, 160, 320, and 640 mAs). The corresponding doses were 0.24, 0.52, 1.1, 2.2 4.4, 8.8, and 17.6 mGy, respectively.

The Barracuda MPD dosimeter calibration coefficient, 1.018, for 80 kVp tube voltage, 2.5 mm Al+0.5 mm Cu, was used to convert the dosimeter reading to the true dose. The calculated expanded uncertainty (2 SD) of the Barracuda MPD using ten averaged samples (80 kVp) was 1.1%. The combined uncertainty ($u_{c}$) was calculated as quadratic summation of uncertainties of Barracuda MPD and MOSFET readout uncertainties (k=2). Furthermore, the lowest acceptable dose and required number of samples to attain 0.3 mGy low‐dose limit were assessed.

The combined uncertainties ($u_{c}$) of photon energy sensitivity measurements were assessed as a weighted sum of variances. These included RADCAL calibration dosimeter expanded (2 SD) energy sensitivity uncertainty 2% (30–70 keV) obtained from RADCAL 1015 datasheet, MOSFET dosimeter calibration factor variation as function of saturation voltage (4%),^(28)^ estimated position induced uncertainty (2%), and cable irradiation uncertainties (1%) according Ehringfelt et al.^(29)^

The MOSFET and TLD point‐dose CBCT measurement combined uncertainty ($u_{c}$) was calculated as the weighted sum of variances including the statistical measurement uncertainty, dosimeter and phantom positioning uncertainties (10%, 10%),^(30)^ MOSFET dosimeter calibration factor variation (4%),^(31)^ X‐ray source variation (5%),^(28)^ and cable irradiation uncertainty (1%).^(29)^

#### D.2 Photon energy sensitivity

**Figure 3:**
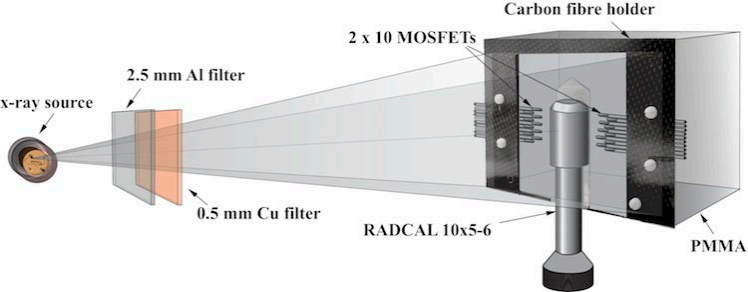
MOSFET dosimeter energy dependency measurement setup with a PMMA backscatter block.

The photon energy sensitivity measurements were performed using 20 MOSFET dosimeters that were attached to an in‐house made carbon fiber frame. The MOSFET positions were carefully chosen as a linear array perpendicular to anode–cathode direction to minimize the heel effect in the anode–cathode direction. Furthermore, the subgroups of ten dosimeters on both sides of the RADCAL $10\times 5^{-6}$ ionization chamber (Fig. 3) were interchanged to compensate for possible variations in dose across the field. The measurements were repeated ten times and an average calibration coefficient (mV/mGy) was calculated. In order to include the backscatter radiation, a 15 cm×15 cm×10 cm thick PMMA block was attached to a carbon fiber holder to simulate soft tissue interactions.^(32)^ The PMMA block dimensions were chosen based on a study by Khan^(33)^ and were, therefore, considered to provide sufficient backscatter for the Cu HVLs (mm) used in this study (Table 1).

The measurements were carried out using a Promax 3D CBCT device operated without gantry rotation and constant doses to minimize possible dose or angular dependency (Al 2.5 mm filter: 17.6±0.2 mGy,Al 2.5 mm+0.5 Cu: 20.6± 0.4 mGy). The tube voltage range was 50–90 kVp with 5 kVp increments. The calculated photon energy spectra and tube voltages (kVp) used are presented in Table 1

MOSFET calibration coefficient (CF), mV/mGy, was determined as the MOSFET reading (mV) divided by the dose measured with the RADCAL 1015 reference dosimeter.^(34)^ Sensitivities for each MOSFET dosimeter were calculated separately. The sensitivity differences between the dosimeters were minimal, and thus the average sensitivity result was finally presented.

#### D.3 Dose dependency

The 20 dosimeters were irradiated ten times on the Promax 3D CBCT device. All exposures were performed using 80 kVp with a 2.5 mm Al filter and seven different mAs values (10, 20, 40, 80, 160, 320, and 640 mAs) over a dose range from 0.24 to 17.5 mGy. The average dosimeter responses were calculated for each dosimeter and dose value.

#### D.4 Dose rate dependency

The dose rate dependency was evaluated by exposing 20 MOSFET dosimeters using a Promax 3D CBCT device (80 kVp, 2.5 mm Al filter, 47.1 keV). All exposures were performed using a constant dose of 16.4 mGy with five different dose rates (0.16, 0.32, 0.67, 1.34, and 2.70 mGy/s). The mean dose rate dependency was calculated for each dosimeter and dose rate.

#### D.5 MOSFET accumulated dose dependency

In order to assess the MOSFET dosimeter accumulated dose sensitivity the following calibration setup was used: 80 kVp, 2.5 mm Al, 47.1 keV, 17.6 mGy, PMMA. The sensitivity of the 20 MOSFET dosimeters was measured after dismantling the dosimeters from the phantom at a 17.500 mV accumulated threshold voltage. The MOSFET sensitivity as a function of the accumulated dose was calculated based on the differences attained at two different threshold voltages: 8300 mV (after the calibration procedure) and 17.500 mV (after dismantling the dosimeters from the phantom).

#### D.6 MOSFET‐TLD point‐dose assessment

MOSFET and TLD comparison measurements were performed using a ProMax 3D MID CBCT device (Fig. 3) (face–maxillofacial protocol, FOV 20 cm×17 cm). All exposures were carried out using 90 kVp tube voltage (2.5 mm Al+0.5 mm Cu filter, 61.1 keV), 6 mA tube current, and 18 s exposure time (108 mAs).

The point doses were obtained using 40 TLDs and 20 MOSFETs that were alternately placed in 20 locations in an anthropomorphic RANDO phantom that represented, according to a previous study,^(28)^ the most radiosensitive organs in the maxillofacial region. Two TLD dosimeters were placed in each anatomic site to calculate the mean value of each specific location. All MOSFET measurements and corresponding read‐outs were repeated ten times to calculate the average dose. Furthermore, all TLDs were exposed ten times and the average dose was calculated from the single read‐out. The dose difference between the MOSFET and TLD dosimeters was calculated as follows: Difference (%)=(MOSFET‐TLD)/TLD.

## III. RESULTS

### A. Uncertainty

**Figure 4:**
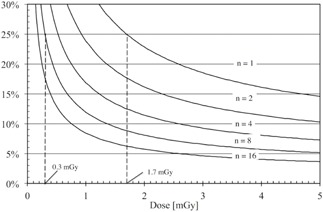
The combined uncertainty (%) of MOSFET dosimeters as a function of dose and number of averaged samples (n=1−16), 1.7 mGy one exposure low dose limit, and TLD comparable (0.3 mGy) low‐dose limit.

The type A standard uncertainty (Error%) was assessed for six dose levels. The uncertainties of the ten repeated measurements were evaluated for each dose using 95% confidence interval (k=2). The combined uncertainty at 95% confidence at different doses were fitted into an exponential function and resulted in the following equation:

(1) $$Err(D)=(0.2853*D^{-0.6924}+0.0518)*n^{-0.5}$$

where *D* is dose in (mGy) and *n* is the number of samples (exposures). The coefficient of determination was $R_{2}=0.99$. The single sample (n=1) low‐dose limit for the MOSFET dosimeter was 1.7 mGy (Fig. 4).

Furthermore, solving Eq. (1) for number of samples (n) and setting dose (D) to the TLD low‐dose limit (0.3 mGy), according to Tarr et al.^(8)^ and Dong et al.,^(9)^ resulted in a total of eight averaged MOSFET samples.

### B. Photon energy sensitivity

The MOSFET sensitivity measured with different filtrations was 3.10±0.10 mV/mGy and 3.08±0.14 mV/mGy using 2.5 mm Al and 2.5 mmAl+0.5 Cu filter combinations, respectively. The photon energy sensitivities (mV/mGy), relative energy sensitivities using 2.5 mm Al filter, and 2.5 mm Al+0.5 mm Cu filter combinations with standard deviation (1 SD) are presented in 5, 6. The MOSFET dosimeter sensitivity was not statistically dependent on the chosen kVp or the filtration according to two‐tailed Student's *t*‐test (p=0.75) and Pearsons correlation $\left(p_{\text{Al}}=0.81,p_{\text{Al}+\text{Cu}}=0.17\right)$.

**Figure 5:**
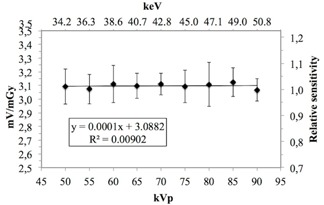
MOSFET dosimeter sensitivity (mV/mGy) using a 2.5 mm Al filter with corresponding error bars (1 SD).

**Figure 6:**
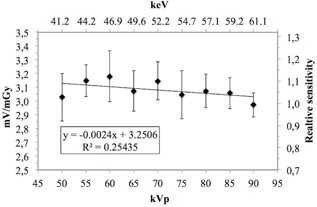
MOSFET dosimeter sensitivity (mV/mGy) using a 2.5 mm Al+0.5 mm Cu filter with corresponding error bars (1 SD).

### C. Dose dependency

The MOSFET dosimeter dose dependency was statistically insignificant with the following linear regression: y=1.003x+0.0396. The MOSFET dosimeters showed a good correlation with the Barracuda MPD reference dosimeter $\left(R^{2}=0.99987\right)$. The standard deviation of the dose dependency measurements ranged from 2.4% to 12.5%.

### D. Dose rate dependency

The MOSFET dosimeter sensitivity was 3.14±0.11 mV/mGy for all evaluated dose rates (0.16–2.70 mGy/s).

### E. MOSFET accumulated dose dependency

Based on the calibration measurements, the average MOSFET dosimeter sensitivity was 3.10±0.10 mV/mGy at 8.300 mV accumulated threshold voltage. The new calibration resulted in 2.83±0.12 mV/mGy sensitivity at 17.500 mV accumulated threshold voltage. The average sensitivity decrease was 9.4% over 9200 mV (17.500‐8.300 mV) threshold voltage variation. The MOSFET sensitivity decrease rate caused by the accumulated dose was 1.02% per 1000 mV.

### F. MOSFET and TLD comparison point‐dose assessment

The MOSFET organ doses ranged between 0.24 mGy and 2.29 mGy and the TLD doses between 0.25 and 2.09 mGy. The mean difference between the MOSFET and the TLD dosimeters was −8%. The average point doses (mGy), differences (%), and the combined uncertainties ($u_{c}$) attained by the dosimeters located in the different layers (no.) of the anthropomorphic phantom using CBCT measurements are presented in Table 2

**Table 2:** Average of ten absorbed dose measurements, difference (%) between MOSFET and TLD values, and combined MOSFET uncertainties

*Organ (layer no*.)	*MOSFET (mGy)*	*TLD (mGy)*	*Difference (%)*	*MOSFET u_c_*
Calvarium anterior (2)	1.25	1.20	3%	18%
Mid brain (3)	1.09	1.36	−20%	18%
Pituitary fossa (3)	1.35	1.29	5%	18%
Right orbit (4)	1.65	2.04	−19%	17%
Right lens (4)	2.29	2.05	12%	16%
Right cheek (5)	1.44	2.06	−30%	17%
Right ramus (7)	1.05	1.54	−32%	18%
Left ramus (7)	1.49	1.98	−25%	17%
Right parotid (6)	1.20	1.45	−17%	18%
Left parotid (6)	1.28	1.66	−23%	18%
Center C‐spine (7)	1.10	1.26	−13%	18%
Left back neck (8)	0.36	0.39	−8%	25%
Right mandible body (7)	1.42	1.41	0%	17%
Left mandible body (7)	1.42	1.62	−12%	17%
Right submandibular gland (8)	1.13	0.52	118%	18%
Left submandibular gland (8)	1.15	0.57	103%	18%
Center sublingual gland (8)	0.46	0.58	−20%	23%
Midline thyroid (9)	0.33	0.32	3%	26%
Thyroid surface (9)	0.24	0.25	−4%	30%
Pharyngeal‐oesophageal space (9)	0.29	0.33	−12%	27%
Average	1.10	1.19		20%

## IV. DISCUSSION

### A. MOSFET dosimeter uncertainty

In the present study, the MOSFET dosimeter uncertainty was evaluated to define the single sample low‐dose limit (25% uncertainty, k=2) and to assess the number of exposures needed to attain the previously proposed TLD (one exposure) low‐dose (0.3 mGy) limit. The purpose of this study was also to assess the MOSFET sensitivity for different energy ranges and to compare the MOSFET point‐dose results with those obtained using TLDs.

According to our study, the TLD comparable lowdose limit (0.3 mGy) can be achieved by averaging eight MOSFET exposures. However, an increase in the number of exposures results in a higher dose and cannot, therefore, be used for *in vivo* dose measurements in order to comply with the ALARA principle (as low as reasonably achievable). Thus, the use of MOSFET dosimeters should only be used for phantom measurements in dentomaxillofacial radiology.

When comparing the single sample low‐dose detection limit of MOSFET dosimeters (1.40 mGy) attained by Yoshizumi at al.^(35)^ with the dose obtained in this study (1.69 mGy), the results showed a 21% higher dose value. The difference in results could be caused by MOSFET sensitivity decrease due to a higher 120 kVp compared to the 80 kVp used in this study. In a prior study performed by Peet and Pryor,^(13)^ the observed minimum (25% total uncertainty, k=2) was 1.5 mGy dose when MOSFET detectors were evaluated for entrance surface dose measurements. Their 11% lower value could have been caused by the water backscatter material which has a lower backscatter factor (BSF) than the PMMA used in this study.

### B. Photon energy sensitivity

We showed that the MOSFET dosimeter sensitivity was independent of the photon energy by using two filter combinations and tube potentials between 50 kVp and 90 kVp. A recent study by Manninen et al.^(21)^ investigated the MOSFET (TN‐1002RD) energy dependency using tube potentials between 40 kVp and 125 kVp. Compared with the reference dosimeter value, they observed a 6% coefficient of variation for tube potentials between 60 and 110 kVp, a −10% decrease at 40 kVp, and a −14.6% decrease at 125 kVp, respectively. These differences can be explained by the lower number of exposures applied (five versus ten), the lower dose (5 mGy versus 17 mGy/20 mGy) affecting the uncertainty, different mean photon energy range (keV), and finally by the backscatter material thickness (10 mm) that was used in their study. Furthermore, another factor that could help explain the differences may have been the different dosimeter attenuation characteristics caused by the reinforcement of the dosimeter type (TN‐1002RD‐H) used in this study.

In an earlier study by Dong et al.,^(9)^ the photon energy response of MOSFET dosimeters were evaluated for skin dose measurements using tube voltages between 40 and 125 kVp $\left(E_{\text{ave}}=26.5–39.7\;\text{keV}\right)$. Their study resulted in a 20% decrease in sensitivity from 40 kVp to 90 kVp. The different energy range and the water backscatter material used by Dong and colleagues may well explain the differences when compared to the statistically insignificant sensitivity variations observed in our study. Furthermore, a study by Peet and Pryor^(13)^ evaluated the energy sensitivity of MOSFETs over a range of 60–100 kVp for diagnostic purposes. The photon energy sensitivity varied between 2.2 mV/mGy–2.9 mV/mGy. The results of their study showed −20% lower sensitivity at 60 kVp compared to the (normalized) 90 kVp value. The lower sensitivity results may have been caused by the different orientation of the MOSFET epoxy bulbs that faced towards the water backscatter material. In our study, the epoxy bulbs were directed towards the X‐ray source to attain the highest sensitivity, based on a previous study by Koivisto et al.^(22)^ Furthermore, Bower and Hinterlang^(19)^ characterized MOSFET dosimeter energy dependency using a (roughly) constant dose and a tube voltage ranging between 40–140 kVp. Their choice of the constant dose, PMMA backscatter material, and RADCAL 1015 reference dosimeter resulted in comparable values to those attained in our study when the results of both studies were normalized to 90 kVp tube voltage.

Even though backscattering is always present in phantom measurements, additional evaluations of the photon energy sensitivities were performed without the PMMA backscatter material (free‐in‐air). Based on the result of the evaluations, the backscatter factor (BSF) could also be determined. The measurements resulted in 17% higher response at 50 kVp compared to the normalized 90 kVp value and in BSF comparable to the findings of Khan.^(33)^ This variation in results indicates that when MOSFETs are used with phantoms, as in our study, the energy dependency should be evaluated using proper backscatter conditions because the scattered X‐rays emphasize the lower energy contribution of the net photon spectrum detected by the MOSFET dosimeter.

### C. Dose dependency

The MOSFET dosimeter dose dependency was evaluated using 0.24–17.5 mGy. The MOSFET dosimeters can be used without dose dependency correction factors for low‐dose (<17.5mGy) measurements. The results were comparable with the findings of Dong et al.^(9)^ and Bower and Hinterlang.^(19)^

### D. Dose rate dependency

The MOSFET dose rate dependency was assessed using 0.16–2.70 mGy/s dose rates. No statistically significant variation in the sensitivity at different dose rates were observed and, therefore, no dose rate correction was needed.^(36)^

### E. MOSFET accumulated dose dependency

The MOSFET sensitivity as a function of the accumulated dose was assessed over a 9.200 mV threshold voltage range. This resulted in a 1% decrease of sensitivity per 1000 mV threshold voltage which is in good agreement with the findings of Brady and Kaufmann^(34)^ and Toncheva et al.^(31)^

### F. MOSFET and TLD comparison

The point doses obtained in the anthropomorphic RANDO phantom using MOSFET dosimeters resulted in a −8% lower average point dose (1.10 mGy) value than that obtained using TLD dosimeters (1.19 mGy). This −8% difference can be explained by a loss of MOSFET sensitivity previously described Brady and Kaufmann^(34)^ (≈1% per 1000 mV) and Toncheva et al.^(31)^ (≈3% per 3000 mV), and by the accumulated (>10.000 mV) average threshold voltage used in the phantom measurements of this study. The dosimeter (1 SD) uncertainties in the phantom measurements ranged between 26% (0.24 mGy) and 7% (2.29 mGy) using ten averaged samples (Table 2). Furthermore, the combined (point dose) (1 SD) uncertainty, calculated as the weighted sum of variances, ranged between 17% and 30%. All TLD and MOSFET point‐dose values, except for those in the left and right submandibular glands, were within the range covered by the extended (2 SD) combined MOSFET uncertainty ($U_{c}$). The higher MOSFET doses observed in the left and right submandibular glands could be due to the vertical position variations of the dosimeters in their designated phantom holes; the surrounding mandible bones may have caused different attenuation and scatter profiles to the dosimeters.

In this study, there were certain limitations. One limitation of the study was that the MOSFET dosimeter performance was not specifically evaluated for clinical applications such as entrance skin dose. Furthermore, the individual MOSFET threshold voltages should have been registered and used for calibration coefficient corrections during the measurements in order to improve the accuracy of the results. When purchasing new dosimeters, calibration is required which subsequently limits the lifespan of the MOSFET dosimeter due to their saturation.

## V. CONCLUSIONS

The MOSFET dosimeters presented statistically insignificant energy dependency for the investigated dental energies. However, the MOSFET dosimeters are not recommended for *in vivo* studies when assessing doses lower than 1.7 mGy since they fail to provide conformance with the target minimum uncertainty of 25% (k2). Nevertheless, the MOSFET dosimeters constitute a feasible dose assessment method for low‐dose (<1.7 mGy) CBCT anthropomorphic measurements using multiple sampling and averaging the results.

## ACKNOWLEDGMENTS

This project was supported by Planmeca Oy. Juha Koivisto is an employee of Planmeca Oy.

## Supporting information

Supplementary Material
