# Supplementary material for: Characterization of MOSFET dosimeters for low‐dose measurements in maxillofacial anthropomorphic phantoms
Source: J Appl Clin Med Phys. 2015 Jul 8;16(4):266–78. doi: 10.1120/jacmp.v16i4.5433 (PMC5690001; doi:10.1120/jacmp.v16i4.5433)
Supplement: Supplementary file 1 — Supplementary Material [file ACM2-16-266-s001.docx]

**Characterization of MOSFET dosimeters for low dose measurements in maxillofacial anthropomorphic phantoms**

Juha Koivisto^1^, Jan Wolff^2^, Timo Kiljunen^3^, Dirk Schulze^4^, Mika Kortesniemi^5^;

^1^Department of Physics, University of Helsinki, Helsinki, Finland; juha.koivisto@planmeca,com

^2^Department of Oral and Maxillofacial Surgery/Oral Pathology, VU University Medical Center, Amsterdam, The Netherlands; jan.wolff@vumc.nl

^3^International Docrates Cancer Center, Helsinki, Finland; timo.kiljunen@docrates.com

^4^Dental Diagnostic Center, Freiburg, Germany; dirk.schulze@ddz-breisgau.de

^5^HUS Helsinki Medical Imaging Center, University of Helsinki, Helsinki, Finland; mika.kortesniemi@hus.fi

^1^Juha Koivisto, *Gustaf Hällströmin katu 2a FI-00560 Helsinki, Finland*

^2^ Jan Wolff, *P.O. Box 7057, 1007 MB Amsterdam, The Netherlands*

^3^Timo Kiljunen, *Saukonpaadenranta 2, FI-00180, Helsinki, Finland*

^4^Dirk Schulze, *Kaiser-Joseph-Strasse 263 79098, Freiburg, Germany*

^5^Mika Kortesniemi, *Haartmaninkatu 4 (POB 340), Helsinki, FI-00029 HUS, Finland*

**Running title:** MOSFET dosimeters in low-dose measurements

**PACS number:** 07.85.Fv, 87.53.Bn, 87.59.B, 87.59.bd, 85.30.Tv
